# Potential for cardiac toxicity with methylimidazolium ionic liquids

**DOI:** 10.1016/j.ecoenv.2022.114439

**Published:** 2023-01-01

**Authors:** Tarek M. Abdelghany, Shireen A. Hedya, Carol De Santis, Sahar S. Abd El-Rahman, Jason H. Gill, Noha F. Abdelkader, Matthew C. Wright

**Affiliations:** a Institute Translational and Clinical Research, Level 4 Leech, Newcastle University, Newcastle Upon Tyne NE2 4HH, United Kingdom; b Department of Pharmacology and Toxicology, Faculty of Pharmacy, Cairo University, Kasr El-Aini St., Cairo 11562, Egypt; c School of Pharmacy, King George VI Building, Newcastle University, Newcastle Upon Tyne NE2 4HH, United Kingdom; d Faculty of Veterinary Medicine, Cairo University, Giza 12211, Egypt; e School of Biomedical, Nutritional and Sport Sciences, Faculty of Medical Sciences, Newcastle University, Newcastle Upon Tyne NE24HH, United Kingdom

**Keywords:** C8[mim], Ionic liquids, Heart, Transporter, MTT, AR42J-B13

## Abstract

Methylimidazolium ionic liquids (MILs) are solvent chemicals used in industry. Recent work suggests that MILs are beginning to contaminate the environment and lead to exposure in the general population. In this study, the potential for MILs to cause cardiac toxicity has been examined. The effects of 5 chloride MIL salts possessing increasing alkyl chain lengths (2 C, EMI; 4 C, BMI; 6 C; HMI, 8 C, M8OI; 10 C, DMI) on rat neonatal cardiomyocyte beat rate, beat amplitude and cell survival were initially examined. Increasing alkyl chain length resulted in increasing adverse effects, with effects seen at 10^−5^ M at all endpoints with M8OI and DMI, the lowest concentration tested. A limited sub-acute toxicity study in rats identified potential cardiotoxic effects with longer chain MILs (HMI, M8OI and DMI) based on clinical chemistry. A 5 month oral/drinking water study with these MILs confirmed cardiotoxicity based on histopathology and clinical chemistry endpoints. Since previous studies in mice did not identify the heart as a target organ, the likely cause of the species difference was investigated. qRT-PCR and Western blotting identified a marked higher expression of p-glycoprotein-3 (also known as ABCB4 or MDR2) and the breast cancer related protein transporter BCRP (also known as ABCG2) in mouse, compared to rat heart. Addition of the BCRP inhibitor Ko143 – but not the p-glycoproteins inhibitor cyclosporin A - increased mouse cardiomyocyte HL-1 cell sensitivity to longer chain MILs to a limited extent. MILs therefore have a potential for cardiotoxicity in rats. Mice may be less sensitive to cardiotoxicity from MILs due in part, to increased excretion via higher levels of cardiac BCRP expression and/or function. MILs alone, therefore may represent a hazard in man in the future, particularly if use levels increase. The impact that MILs exposure has on sensitivity to cardiotoxic drugs, heart disease and other chronic diseases is unknown.

## Highlights

• The MIL - M8OI [C8mim] - has been found in the environment and in man.
• MILs affect rat cardiomyocyte beat rate, beat amplitude and cell survival in vitro.
• Cardiac injury is seen in rats treated with MILs.
• Species difference for cardiac toxicity between rats and mice likely with MILs.
• Murine resistance to cardiac toxicity may be due in part to cardiac Bcrp.

## 1 Introduction

Ionic liquids are organic salts that, due to their ionic structure, have low volatility. This property can make them useful solvent molecules since they are often liquids at ambient temperatures, providing solutions to problematic issues, such as the solvation of drugs and therapeutics (Stoimenovski et al., 2010, Wang et al., 2018, Ossowicz et al., 2019). Ionic liquids can be constituted of relatively innocuous chemicals such as amino acids (Kirchhecker and Esposito, 2016, Davis, 2003). However, several classes of man-made ionic liquids have been developed, such as the MILs, a class that is finding increasing use in a variety of industrial processes. These include electrochemical applications, petroleum alkylation, storing and transporting of toxic gases, hydrogenation processes, use in anti-statics, natural fibre dissolution and use as heat transfer materials and lubricants (Ostadjoo et al., 2018, Leitch et al., 2020a, Greer et al., 2020). Drivers remain for their continued increased use since ionic liquids in general may play an important role in environmental protection (Malolan et al., 2021, Schuur et al., 2019) and carbon capture/reduced global warming (Krishnan et al., 2020).

The MIL class of ionic liquids comprises at least 60 variants based on registrations with the European Chemicals Agency (ECHA). Each variant differs in the length of the N-linked alkyl chain in the methylimidazolium cation [2 C alkyl chain, 1-ethyl-3-methylimidazolium (EMI); 4 C alkyl chain, 1-butyl-3-methylimidazolium (BMI); 6 C chain, 1-hexyl-3-methylimidazolium (HMI); 8 C chain, 1-ocytl-3-methylimidazolium (M8OI) and 10 C alkyl chain, 1-decyl-3-methylimidazoium (DMI)] and/or the anion used within the salt (for a review, see Leitch et al., 2020a). For these studies, the Cl^-^ salts were selected for study (rather than those with more complex anions) in order to solely study the potential toxic effects of the MIL cation moiety.

There are limited data overall on the stability of MILs in the environment. The evidence to date suggests that the MILs may be relatively persistent in the environment (Modelli et al., 2008, Liwarska-Bizukojc et al., 2015, Wang et al., 2017, Oskarsson and Wright, 2019). Further, although there may be metabolic action on the longer chain alkyl groups in the region furthest from the methylimidazolium moiety, the presence of the imidazolium ring (which does not exist in nature), may significantly inhibit degradation of short alkyl chain variants and prevent complete degradation of any MIL to common naturally-present breakdown products (Liwarska-Bizukojc et al., 2015).

Recent work from this laboratory identified a substance in soil around an urban landfill site that was toxic to a rat liver progenitor (B-13) cell line, subsequently identified as being associated with M8OI (also known as C8[mim]) (Probert et al., 2018, Leitch et al., 2020a). It was determined that the hepatic carboxylic acid metabolite of M8OI was capable of being incorporated into the autoantigen associated with the auto-immune liver disease primary biliary cholangitis (PBC) and therefore we proposed that M8OI and other MILs are potential hazard triggers for the disease. More recently, M8OI has been shown to be detectable in human sera (Leitch et al., 2021). Whilst these observations do not establish a cause and effect with regard to PBC, it does indicate that both the environment and human population are being exposed to M8OI, and presumably other MILs (particularly smaller alkyl chain variants, which have higher use levels in the EU (Leitch et al., 2020a).

The toxicity of MILs to environmental species have been most widely examined and in general, data overall suggest that if levels accumulate above a variable threshold, adverse effects are observed in multiple environmental model systems (for a review of M8OI, see Leitch et al., 2020a). However, potential toxicity of MILs in mammalians has received little attention to date.

Addition of MILs to neural and hepatic cells in culture have identified mitochondria as the key target for the initiation of an apoptotic mode of cell death (Li et al., 2012, Ma and Li, 2018, Probert et al., 2018). Toxicity in hepatic cells is heavily influenced by the length of the alkyl chain, with longer alkyl chain MILs being more potent (Abdelghany et al., 2020). For example, in the liver progenitor B-13 cell line, alkyl chain length accounts for > 700-fold difference in potency between 2 C and 10 C alkyl chain MILs (Abdelghany et al., 2020). The increased MIL toxicity seen with increased alkyl chain length is associated with an increased propensity to form micelles in aqueous media and subsequent inhibitory interactions with membrane-associated mitochondrial electron transport (Abdelghany et al., 2020).

Limited acute toxicity studies in mice with M8OI using an intraperitoneal route of exposure have identified that either the liver (Yu et al., 2008) or the kidney (with some liver changes) are targets (Leitch et al., 2020b). A single - longer term (18 weeks) oral study in mice with M8OI or BMI - also identified the kidney as a target organ, and showed that both chemicals altered the gastrointestinal microbiota (Young et al., 2020). None of the studies in mice observed toxic effects of MILs in heart tissue. However, the heart is likely to be an important target for environmental chemical toxicity. In this respect, cardiovascular toxicity is known to be a major cause of drug attrition, reported to have led to 27.3% of drugs being dropped from development between 1993 and 2006 (Guengerich, 2011).

In this study, the effects of MILs on rat neonatal cardiomyocyte function were initially examined in vitro. Cardiomyocytes are mononucleated, organised into myofilaments and constitute roughly 32% of the total number of heart cells in humans and rodents (Bergmann et al., 2015). They are key resident cells of the myocardium and form the heart’s contractile core. They have limited regenerative capacity in vivo and their injury severely undermines the physiological functionality of the heart. The direct electrical contact between individual myocytes and other cardiac cells is achieved through a distinct pattern of cell-cell interactions, gap junctions and ion channels (Netter, 2014). We show for the first time that MILs induce changes in rat neonatal cardiomyocyte beat rate and amplitude prior to a loss of viability, with increased severities seen with longer chain MILs. We demonstrate that the heart is a target organ in the rat for MIL toxicity in vivo and that BCRP expression in mouse heart may in part, protect them from the cardiotoxic effects of MILs.

## 2 Material and methods

### 2.1 Materials

All 1-alkyl-3-MILs (as the Cl^-^ salt, > 96% purity) were purchased from Sigma (Poole, UK):- EMI C_6_H_11_ClN_2_ (CAS # 65039–09–0); BMI C_8_H_15_ClN_2_ (CAS # 79917–90–1); HMI C_10_H_19_ClN_2_ (CAS # 171058–17–6); M8OI C_12_H_23_ClN_2_ (CAS # 64697–40–1) and DMI C_14_H_27_ClN_2_ (CAS # 171058–18–7). The Cl^-^ salts were selected for study (rather than those with more complex anions) in order to solely study the potential toxic effects of the MIL cation moiety. Oligonucleotides were custom synthesised by Sigma (Poole, UK). For antibodies, see supplementary data.

### 2.2 Cardiomyocyte isolation and culture

Primary cardiomyocytes were prepared from whole neonatal rat hearts. Twelve to twenty-four hours after birth, rat pups were euthanised by decapitation and dissected to isolate heart tissue. The upper portion of the heart - including residual portions of the aortic arc, valves and atria - were removed and washed with Hank’s balanced salt solution (0.14 M NaCl, 5.4 mM KCl, 0.34 mM Na_2_HP0_4_0.7 H_2_0, 0.44 mM KH_2_P0_4_, 5.6 mM D-glucose and 15.7 mM NaHC0_3_) to eliminate blood clots and residues, prior to mincing (1–2 mm^3^ pieces) in a sterile petri dish using sterile surgical scissors. The minced tissue was suspended in equal volumes of Dulbecco’s Minimal Essential Medium (DMEM) and trypsin-EDTA and incubated in a shaking incubator at 37 °C under normoxic conditions for 40–45 min. The resultant digested tissue was passed through a 100 µm sterile mesh cell strainer (Falcon) and the filtrate centrifuged at 500 g for 5 min at room temperature. The supernatant was discarded and the resulting cell pellet was re-suspended in DMEM supplemented with 10% (v/v) FCS and cultured in plastic tissue culture dishes at 37 °C in an humidified incubator at 5% CO_2_ under for 1 h to allow fibroblasts to adhere to the substratum. The fibroblast-depleted suspension was then transferred to a culture flask pre-coated with 0.5% fibronectin in 0.02% bovine gelatine and returned to the incubator.

### 2.3 xCELLigence studies

The xCELLigence Cardio instrument (ACEA Biosciences, Agilent, USA) was used to determine both overall culture-generated and rapid (12.9 ms) changes in impedance. A typical experiment essentially followed the manufacturer’s instructions. In brief, a Cardio E-plate (ACEA Biosciences) was pre-coated with a bovine gelatin/fibronectin matrix (0.5%/0.02%). Prior to seeding with cardiomyocytes, a background scan was performed. Cardiomyocytes were then seeded DMEM supplemented with 10% (v/v) FCS and incubated at room temperature for 30 min to allow an even deposition of the cells onto the electrode surface. The E-plate was then inserted in the Cardio system cradle housed in an humidified incubator at 37 °C and 5% CO_2_. Duration and frequency of impedance recordings were selected and modified using xCELLigence RTCA Cardio software version 1.2. The cells were cultured in the E-plate with medium change every 48 h.

The viability and function of cardiomycytes were determined through continuously monitoring overall culture-generated impedance and the rapid changes in impedance associated with beat rate and beat amplitude respectively. Beat rate was calculated as the number of positive peaks per minute. Beat rate changes were double-normalised (∆∆) to baseline and vehicle control and expressed as percentage change. Beat amplitude was calculated as whole peak counts from each negative peak to the following positive peak, and are expressed as a percentage change, double-normalised to baseline and vehicle control.

### 2.4 Cell line culture and viability

The murine atrial cardiomyocyte HL-1 cell line (Claycomb et al., 1998) was routinely cultured in Claycomb medium (Sigma, Poole UK) containing 10% (v/v) fetal calf serum (FCS), 80 units/ml penicillin, 80 μg/ml streptomycin and 0.584 g/l l-glutamine. HL-1 cells were sub-cultured after washing cells with HBSS and incubation with trypsin-EDTA solution (0.05% trypsin in 0.02% EDTA-Na) (Sigma, Poole, UK). An equal volume of soybean trypsin inhibitor was added to inactivate trypsin digestion and the cells were harvested in FCS- and norepinephrine-free Claycomb medium, centrifuged at 500 g for 5 min and cell pellets resuspended and cultured in complete Claycomb medium. Media was replenished daily. Rat B-13 cells were cultured in low glucose (1000 mg/L) Dulbecco’s Modified Eagle’s Medium (DMEM) containing 10% (v/v) fetal calf serum (FCS), 80 units/ml penicillin, 80 μg/ml streptomycin and 0.584 g/l l-glutamine. For a review of the cell line, see Probert et al. (2015). All cell lines were incubated at 37 °C in a humidified atmosphere of 5% CO_2_ in air. For additional information regarding the MTT reduction as a proxy for cell viability, see supplementary data.

### 2.5 Animal studies

These studies were performed according to the principles of the Care and Use of Laboratory Animals published by the US National Institutes of Health (NIH Publication No. 85–23, revised 2011) and granted approval with permit number (PT 2337) from the Ethics Committee for Animal Experimentation, Faculty of Pharmacy, Cairo University. Ethical approval from the Animal Welfare Ethical Review Board at Newcastle University was also obtained.

Male Wistar rats originally bred at the animal facility of Faculty of Pharmacy, Cairo University, Cairo, Egypt were housed and used at the same facility over the study period. Rats were kept in an air-conditioned environment of temperature (25 ± 2 °C), humidity (60 ± 10%) and a 12 h day/night cycle with free access to food and water ad libitum throughout the study.

For the acute study, 18 male Wistar rats were allocated in random to six groups. As a qualitative study, only 3 animals per group were employed in order to reduce the numbers used whilst retaining the option to apply statistical tests on endpoints. Only male rats were used to further reduce the number of animals used and to avoid potential complications of variations in sex hormone levels seen in females. Rats were administered ionic liquid chemicals dissolved in sterile phosphate buffer saline (137 mM NaCl, 27 mM KCl, 100 mM phosphate pH 7.4, PBS) at either 80 mg/ml (EMI, BMI) or 30 mg/ml (HMI, M8OI, DMI) or PBS vehicle control by intraperitoneal injection at 0, 24, 48,72 and 90 h prior to cervical dislocation at 96 h. To avoid potential interactions of ionic liquid counter-ion toxicities, only MIL chloride salts (i.e. the common physiological anion) were used. To reduce the number of animals used in this study, only a single dose was employed for each compound. The doses chosen for each ionic liquid (80 mg/kg body weight for EMI and BMI; 30 mg/kg body weight for HMI, M8OI and DMI) were selected based on data showing that increased alkyl chain length was associated with increased toxicity in vitro (Abdelghany et al., 2020) and that renal and hepatic effects have been observed after intraperitoneal administration of M8OI within the dose range 10–40 mg/kg body weight (Yu et al., 2008, Leitch et al., 2020b). This short-term systemic exposure protocol was adopted in order to maximise detection of target organ effects whilst reducing the number of animals used and reducing potential for severe adverse effects.

For the chronic study, 28 male Wistar rats (180–220 g body weight) were randomly allocated into 8 cages (3–4 rats per cage) and every two cages were randomly assigned to one of the treatment four groups (2 cages per group, 7 rats per group) provided with either normal tap water; tap water containing HMI; tap water containing M8OI or tap water containing DMI. Rats were treated with MILs at a concentration of 880 mg/L for 20 weeks (equivalent to 44 mg/kg bw per day (EFSA Scientific Committee, 2012). This dose was selected since mice readily tolerated M8OI in drinking water at 440 mg/L (Young et al., 2020) and a preliminary study in rats with M8OI (0, 220, 440, 880 mg/L drinking water for 4 weeks) was readily tolerated at the highest dose. Since M8OI was determined to stable in water for at least one month (Young et al., 2020), fresh drinking water stocks were prepared each month.

### 2.6 Clinical chemistry

Tail vein blood was collected and serum prepared. Serum troponin levels were determined using a kit supplied by Roche using a Cobas E 411 analyser (Roche). Serum CK-MB levels were determined using a kit supplied by Beckman Coulter using an AU480 Chemistry Analyzer (Beckman Coulter).

### 2.7 Tissue pathology

Immediately after sacrifice by cervical dislocation, tissues were removed and fixed in 10% formalin in PBS, processed, embedded in wax and sections stained with haematoxylin and eosin essentially as previously outlined (Meyer et al., 2017).

### 2.8 qRT-PCR

Surplus adult (> 10 weeks of age) male rats and mice (C57Bl6) made available from other studies were killed by cervical dislocation. Total RNA was isolated from various tissues using Trizol essentially according to the manufacturer’s instructions, with integrity and quantification accomplished through agarose gel electrophoresis and UV spectrophotometry as described (Probert et al., 2014).

Relative transcript abundances were determined by qRT-PCR using Sybrgreen and primer pairs designed to specifically hybridise to conserved sequences present in the rat and mouse transcripts. Where possible, primer pairs amplified a PCR product that was separated by at least one intron on the corresponding genomic DNA of the species used for identifying appropriate hybridisation sites (primer melting temperatures 57–63 °C, 60 °C as optimum; PCR product size 50–80 bp where possible; at least 4 mismatches with unintended targets). See supplementary Table 1 for details.

### 2.9 Western blotting

Protein extracts were prepared from various tissues by homogenising in ice-cooled RIPA buffer (30 mM HEPES, 150 mM NaCl, 1% (w/v) Nonidet P-40, 0.5% (w/v) sodium deoxycholate, 0.1% (w/v) sodium dodecyl sulfate and 5 mM EDTA) and centrifugation to remove clumps. Aliquots were snap frozen and protein concentrations later determined using the Lowry method. Western blotting was performed essentially as outlined (Marek et al., 2005), followed by visualisation using an ECL kit and x-ray film. Expression relative to beta-actin was determined by image analysis using Image J.

### 2.10 Statistics

Data are expressed as means ± standard deviation (SD) unless otherwise indicated. Comparisons between means were carried out using the Student’s T test for 2 group comparisons and one way analysis of variance (ANOVA) test for multiple group comparisons (when appropriate), followed by a Bonferroni multiple comparison’s tests. For all statistical tests, the level of significance was fixed at p < 0.05 (two tailed). Kruskal Wallis H test was used for comparing the frequency data for nonparametric analyses, followed by the Mann-Whitney *U* test.

## 3 Results

### 3.1 MILs are toxic to rat cardiomyocytes in vitro

**Fig. 1:**
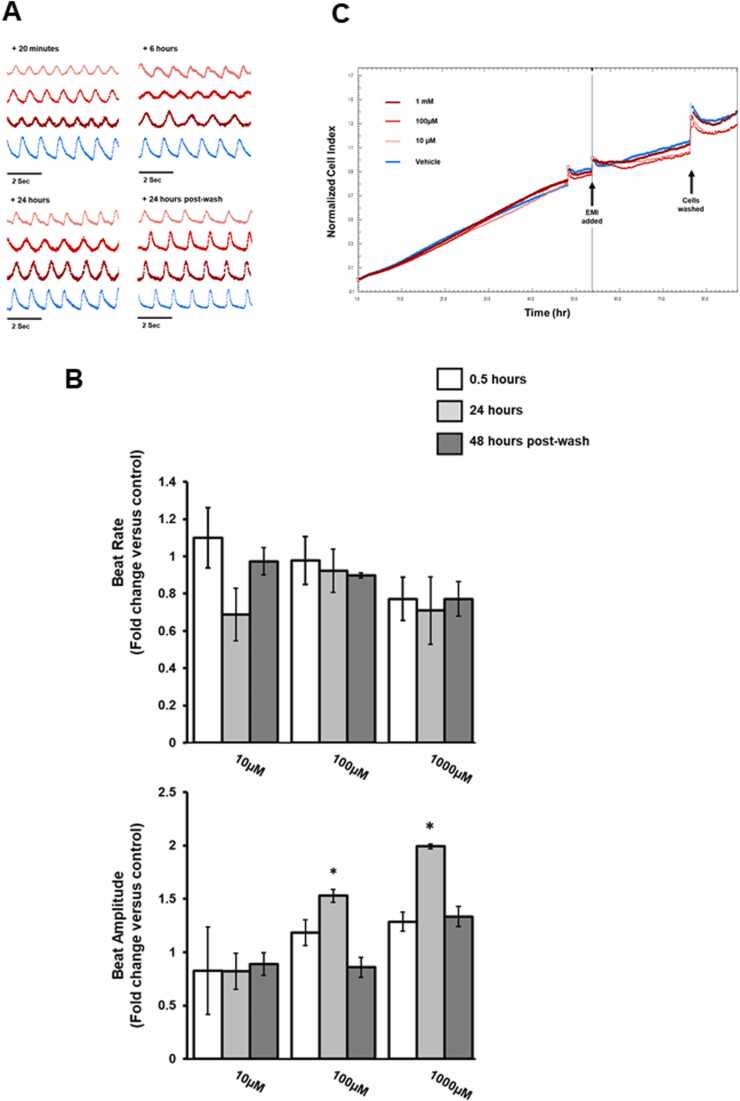
Effect of EMI on rat cardiomyocyte function and viability in vitro. A, representative traces showing changes in cell contractility following exposure to EMI at the indicated concentration and timepoints after addition. B, changes in beat rate and beat amplitude are represented as fold changes relative to control. Results are the mean of at least 3 separate determinations from the same experiment ± SD, *p < 0.05. C, xCELLigence trace showing cell index (indicative of cell survival) over time. Data plot is normalised to point of compound addition. All results typical of at least 3 separate experiments.

Rat neonatal cardiomyocytes were chosen as an in vitro model in which to screen for potential cardiotoxic effects of MILs since they provide a practical model to examine contraction, ischaemia, hypoxia and toxicity (Chlopcíková et al., 2001). Fig. 1 demonstrates that EMI did not affect cardiomyocyte beat rate up to a concentration of 1 mM but resulted in significant changes in beat amplitude at 100 µM and 1 mM after 24 h exposure. Based on the normalised cell index, there was no loss of cell viability over the timecourse of the experiments.

**Fig. 2:**
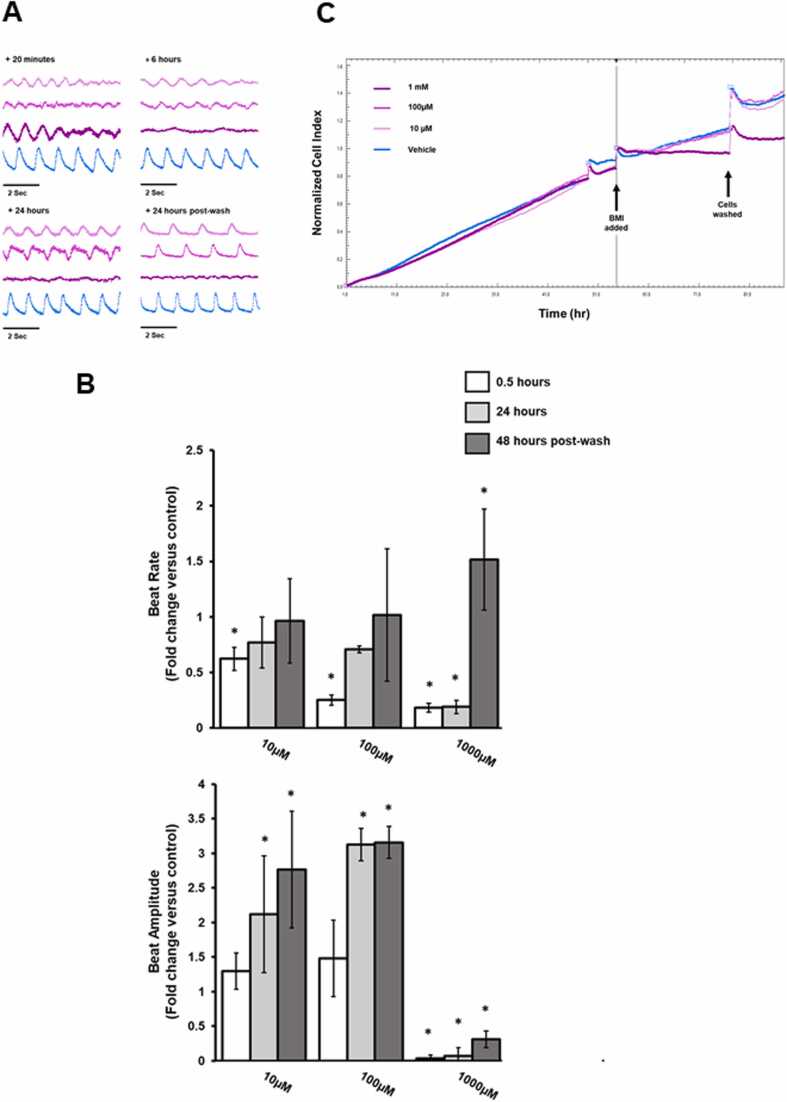
Effect of BMI on rat cardiomyocyte function and viability in vitro. A, representative traces showing changes in cell contractility following exposure to BMI at the indicated concentration and timepoints after addition. B, changes in beat rate and beat amplitude are represented as fold change relative to control. Results are the mean of at least 3 separate determinations from the same experiment ± SD, *p < 0.05. C, xCELLigence trace showing cell index (indicative of cell survival) over time. Data plot is normalised to point of compound addition. All results typical of at least 3 separate experiments.

Fig. 2 demonstrates that BMI treatment resulted in significant reductions in beat rate at all concentrations (10 µM, 100 µM and 1 mM) within 30 min, followed by a recovery to near control rates in cells treated with 10 µM and 100 µM at 24 h and 48 h post compound removal. The beat rate in 1 mM-treated cells 48 h post compound removal was significantly greater that control. Beat amplitude over this period significantly changed at all concentrations by 24 h, significantly increased at 10 µM and 100 µM but was virtually abolished at 1 mM. These changes did not reverse 48 h post compound removal at any treatment level. Based on the normalised cell index, there was a loss of cell viability over the time course of the experiments in cells treated with 1 mM BMI.

**Fig. 3:**
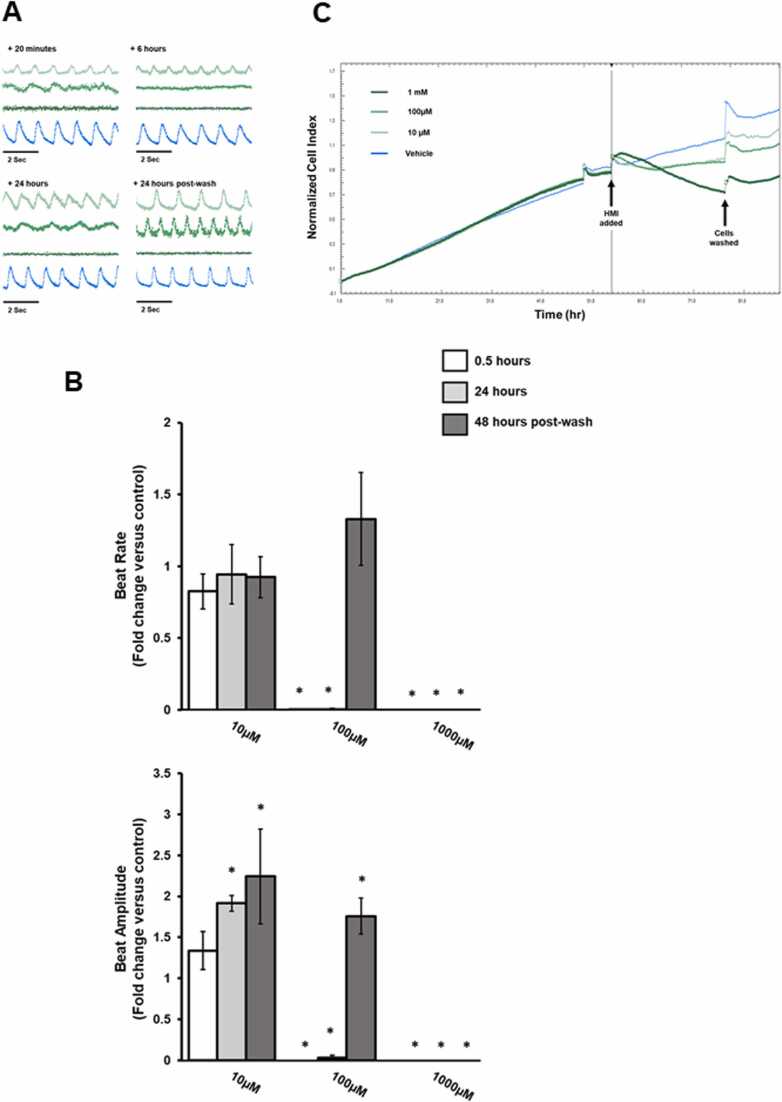
Effect of HMI on rat cardiomyocyte function and viability in vitro. A, representative traces showing changes in cell contractility following exposure to HMI at the indicated concentration and timepoints after addition. B, changes in beat rate and beat amplitude are represented as fold change relative to control. Results are the mean of at least 3 separate determinations from the same experiment ± SD, *p < 0.05. C, xCELLigence trace showing cell index (indicative of cell survival) over time. Data plot is normalised to point of compound addition. All results typical of at least 3 separate experiments.

The effects of 1-hexyl-3-methylimidazolium (HMI) are shown in Fig. 3. HMI treatment at 10 µM had little effect on cardiomyocyte contractility whereas 100 µM and 1 mM abolished beating within 30 min. Beating was returned to normal after washout of 100 µM but remained absent in cardiomyocytes treated with 1 mM. Despite having no effects on beat rate, 10 µM HMI induced a statistically significant increase in beat amplitude. This effect was not reversed by washout. Beat amplitude was abolished by 100 µM and 1 mM HMI within 30 min and as for beat rate, only washout of 100 µM resulted in a recovery in beat amplitude, although at a level significantly higher than control cells. HMI caused significant changes in beat rate within 30 min exposure at 100 µM and 1000 µM. Overall contractility stopped at higher concentrations, resumed after 100 µM compound removal but not after removal of 1000 µM. Beat amplitude increased at 10 µM 24 h post exposure. Based on the normalised cell index, there was a loss of cell viability over the timecourse of the experiments at all concentrations, with higher concentrations of HMI leading to greater loss of viability.

**Fig. 4:**
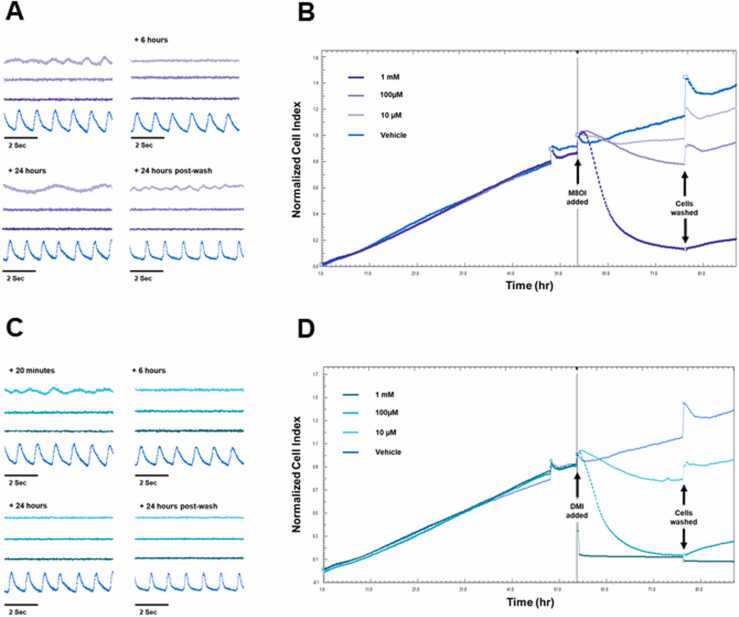
Effect of M8OI and DMI on rat cardiomyocyte function and viability in vitro. A, representative traces showing changes in cell contractility following exposure to M8OI at the indicated concentration and timepoints after addition. B, xCELLigence trace for M8OI showing cell index (indicative of cell survival) over time. Data plot is normalised to point of compound addition. C, representative traces showing changes in cell contractility following exposure to DMI at the indicated concentration and timepoints after addition. D, xCELLigence trace for DMI showing cell index (indicative of cell survival) over time. Data plot is normalised to point of compound addition. All results typical of at least 3 separate experiments.

M8OI is a MIL that was recently detected in the environment (Probert et al., 2018, Leitch et al., 2020a). Fig. 4a and b demonstrate that cardiomyocyte treatment with M8OI resulted in severe effects on contractile function within 20 min at all concentrations, precluding a determination of beat rate and amplitude. These effects were not readily reversed, except for a limited return of contractility after washout of 10 µM M8OI. Cell viability was markedly affected in a dose-responsive manner, with 1 mM M8OI leading to marked cell detachment from the culture vessel.

Fig. 4c and d indicate that cardiomyocyte exposure to DMI resulted in the most severe effects on contractile function, with a loss of any regular contractility within 20 min at all concentrations. This response also precluded a determination of beat rate and amplitude. These effects of DMI were not reversed by washout. Cell viability was markedly affected in a dose-responsive manner, with both 100 µM and 1 mM DMI treatments leading to marked cell detachment from the culture vessel.

These data demonstrate that MILs are toxic to rat neonatal cardiomyocytes in vitro in a time and dose-dependent manner and that increased toxicity is observed with increasing alkyl chain length as summarised in supplementary data Table 2. The changes in beat rate and beat amplitude observed to occur in response to MIL exposure to rat neonatal cardiomyocytes are to be likely associated with a combination of effects on mitochondrial function (a key initiating event in B-13 cell death (Abdelghany et al., 2020)), adverse membrane effects and an impairment of ionic current flows. In this respect, it is known that MILs are capable of interfering with membrane permeability and biophysical characteristics (Hayakawa et al., 2013, Jing et al., 2014).

### 3.2 MILs are cardiotoxic to rats in vivo

**Fig. 5:**
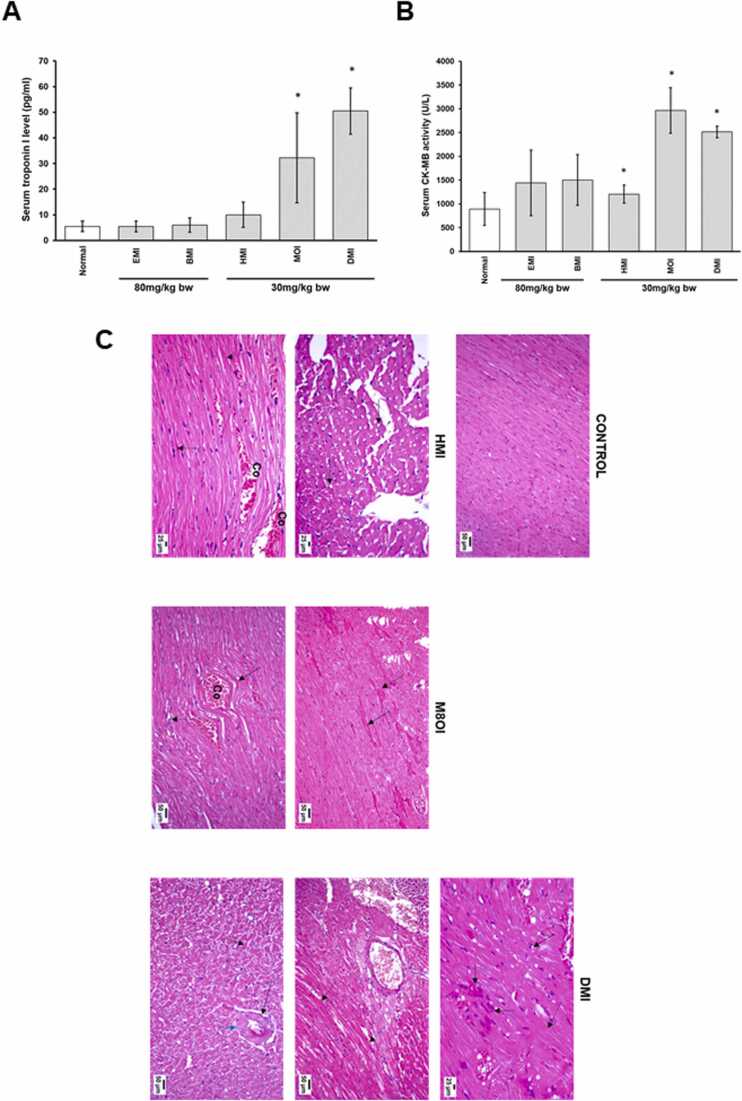

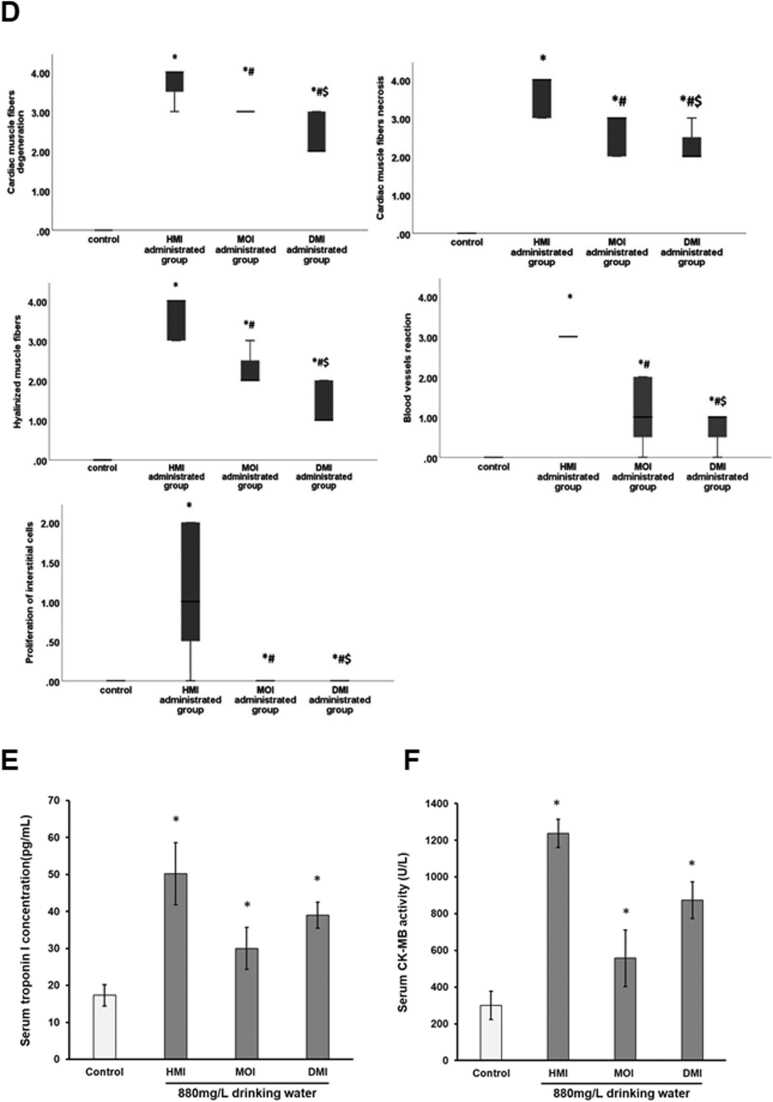
Effect of MILs on cardiac injury in rats. Adult male rats (3 per group) were treated daily by intraperitoneal injection with PBS (vehicle control) containing additionally the indicated MIL at the dose indicated. Blood samples were collected 6 h after the last dose. A) Serum troponin I and B) serum CK-MB levels at sacrifice. Data are the mean and SD of 3 animals per group. *Statistically significantly different (two tailed, p < 0.05) from control using the one way analysis of variance (ANOVA) test followed by Bonferroni multiple comparison’s tests. C, adult male rats (7 per group) were exposed to drinking water (control) containing additionally 880 mg/L of a MIL for 5 months, prior to sacrifice and examination of tissues: photomicrograph of H&E-stained heart sections of; (i) control rat showing normal histological structure, (ii and iii) HMI treated rat showing diffuse vacuolar degeneration (arrow) of the cardiac muscle fibres, scattered hypereosinophilia (dashed arrow), and congestion of the coronary blood vessels (Co); (iv and v) MOI treated rat showing cardiomyocyte degeneration and increased hypereosinophilia (arrow), foci of macrophages phagocytosing the debris of the necrotic fibres (dashed arrow), and congestion of the coronary vessels (Co); (vi-viii) DMI treated rat showing vacuolization of the myocardial muscle fibres (arrow), hyalinization (dashed arrow), aggregation of macrophages engulfing necrotic debris, congestion (Co), and coronary vessel wall hyalinization (blue arrow) and perivascular oedema (thin arrow). D, severity of histopathological lesions were scored by a pathologist blinded to the treatment groups using a scale of 0–4 where “0” denotes absence of the histopathological lesion in the examined heart section; “1” signalizes that the distribution of the observed lesion is up to 25% of the area examined; “2” signalizes that the distribution of the observed lesion is between 26% and 50% of the area examined; “3” signalizes that the distribution of the observed lesion is between 51% and 75% of the area examined; and “4” signalizes that the distribution of the observed lesion is more than 75% of the area examined. These non-parametric data are presented as median (max-min) and were analysed using the Kruskal Wallis H test followed by the Mann-Whitney U test. *Significantly different (two tailed, p < 0.05) when compared to control group; ^#^significantly different (two tailed, p < 0.05) when compared to HMI group; ^$^significantly different (two tailed, p < 0.05) when compared to MOI. E, serum troponin and F, serum CK-MB levels. Data are the mean and SD of 7 animals/group. *Significantly different (two tailed, p < 0.05) from control using the one way analysis of variance (ANOVA) test followed by Bonferroni multiple comparison’s tests.

In order to determine whether the heart is likely to be a target organ for the toxic systemic effects of MILs, a limited single dose acute toxicity study using an intraperitoneal route of administration was performed in rats. Fig. 5a and b indicate that high systemic levels of the longer alkyl chain MILs had marked effects on cardiac tissue based on significant increases in serum levels of biomarkers for cardiac injury troponin 1 and CK-MB activity.

Although this limited acute toxicity study indicates a potential for cardiac toxicity in rats, MILs are water-soluble, persist in the environment and an oral route of exposure is a more realistic exposure scenario for the general population. Furthermore, absorption from the gastrointestinal tract and systemic availability are predicted to be significantly influenced by the length of the alkyl chain in that the shorter chain variants are predicted to be more readily absorbed (Young et al., 2020). Accordingly, the effects of exposing rats via drinking water to the 3 longer alkyl chain MILs (HMI, M8OI and DMI) was examined.

The heart of control rats had normal histological structure, orientation and striation of the cardiac muscle fibres while heart tissue sections from rats treated with the longer chain MILs showed increasing degrees of cardiomyopathy with severity increasing with increasing alkyl chain length (Fig. 5c-f). The heart tissue of HMI-treated rats revealed diffuse vacuolar degeneration of the cardiac muscle fibres and scattered hypereosinophilia with a moderate degree of inter-muscular oedema and necrosis (Fig. 5c-f). Congestion of the coronary blood vessels was evident (Fig. 5c-f). The MOI-treated rats showed degeneration and necrosis of the cardiac muscle fibres with increased hyper-eosinophilic structure-less hyalinized muscle fibres (Fig. 5c-f). Rare foci of macrophages phagocytosing the debris of the necrotic fibres was also observed (Fig. 5c-f). Some rats showed coronary vessel congestion and mild inter-muscular oedema. The hearts of DMI-treated rats revealed diffuse myocardial muscle fibre degeneration, vacuolization and necrosis. In addition, hyalinization was evident as indicated by diffuse bright eosinophilic fibres with shrunken or fragmented nuclei and loss of muscular striation (Fig. 5c-f). Focal areas of phagocytosing macrophage aggregation within necrotic debris was observed. The coronary blood vessels in the vicinity revealed congestion (Fig. 5c-f) and perivascular oedema with occasional focal areas of wall hyalinization (Fig. 5c-f).

These cardiac changes occurred alongside evidence of kidney injury (data not included), as previously observed also in mice although in the absence of cardiac effects (Leitch et al., 2020b).

### 3.3 Expression of BCRP is a determinant in (murine) cardiac toxicity

Previous work in mice identified that the kidney was the target organ for M8OI toxicity, accompanied by effects in the liver (Leitch et al., 2020b, Young et al., 2020). No adverse effects were observed in mouse heart tissues. From extensive studies into drug efficacy and toxicity, it is widely acknowledged that the proteins transporting drugs into and out of cells are important determinants (DeGorter et al., 2012). Since M8OI would be expected to be fully ionised to its M8OI^+^ cation and Cl^-^ anion in aqueous solution and the Oct (Slc22) family of transporters encodes proteins that are electrogenic (Na+-independent) transporters of organic cations, this family was considered a likely candidate for M8OI uptake (Hedya et al., 2023). There are 3 organic cation transporter genes in rats: Oct1 (Slc22a1), Oct2 (Slc22a2) and Oct3 (Slc22a3) and the rat OCT1 inhibitor clonidine (Floerl et al., 2020) inhibited M8OI toxicity in B-13 cells (Hedya et al., 2023). Accordingly, the expression of OCTs in mouse and rat heart tissue was examined. The OCT1/3 inhibitor quinidine (Chen et al., 2020) unexpectedly inhibited M8OI toxicity in B-13 cells (Hedya et al., 2023). However, quinidine is a substrate for p-glycoproteins in the rat (Kusuhara et al., 1997, Mori et al., 2012). The expression of p-glycoproteins and a transporter commonly associated with drug resistance – breast cancer related protein (BCRP) – were also examined in mouse and rat heart tissue. Complementary studies have also demonstrated that M8OI inhibits the excretion of the fluorescent p-glycoproteins and BCRP substrate Hoechst 33342 from rat B-13 cells. However, only p-glycoproteins substrates/inhibitors exacerbate the toxicity of M8OI in rat B-13 cells in contrast to the BCRP inhibitor Ko143, suggesting that in rats, p-glycoproteins are a major route for transport of M8OI out of the cell (Hedya et al., 2023). M8OI toxicity in B-13 cells is also modulated (inhibited) by OCT1 inhibition, although less effectively than p-glycoproteins substrates/inhibitors, suggesting that uptake may not be fully selective (Hedya et al., 2023). The higher expression of p-glycoprotein-1 in B-13/H cells may also explain their 10-fold reduced sensitivity to M8OI compared to B-13 cells (Abdelghany et al., 2020).

It was therefore hypothesised that the difference between rat and mouse and their sensitivities to MIL cardiac toxicity is associated with differences in the expression of either transporters mediating uptake (e.g. OCTs) and/or transporters mediating excretion (e.g. p-glycoproteins, BCRP). To test this hypothesis, the role of i) p-glycoproteins in the excretion of other MILs (EMI, BMI, HMI and DMI) in rat B-13 cells and ii) the expression of transporters were compared between rat and mouse heart.

Figure S1 demonstrates (with HMI) the typical effect seen when the p-glycoproteins inhibitor cyclosporin A is co-incubated with longer chain MILs in that the toxicity of the MIL is increased in rat B-13 cells. Table S3 summarises the data seen with all MILs and overall suggests that HMI, M8OI and DMI – but not EMI and BMI - are primarily substrates and are excreted from cells via p-glycoproteins in rat.

The expression of p-glycoproteins (*Abcb1b and Abcb4*) and *Bcrp* transcripts relative to their *18 S* rRNA levels was initially examined in rat and mouse tissues by qRT-PCR. To ensure comparability, the same primer pairs were used to amplify PCR products for orthologous transcripts (i.e. using appropriate regions of cDNA sequence sharing 100% sequence identity). All qRT-PCR stages were performed at the same time under the same conditions so that any differences in apparent relative transcript expression were likely to be reflective of real differences in relative expression between rat and mouse.

**Fig. 6:**
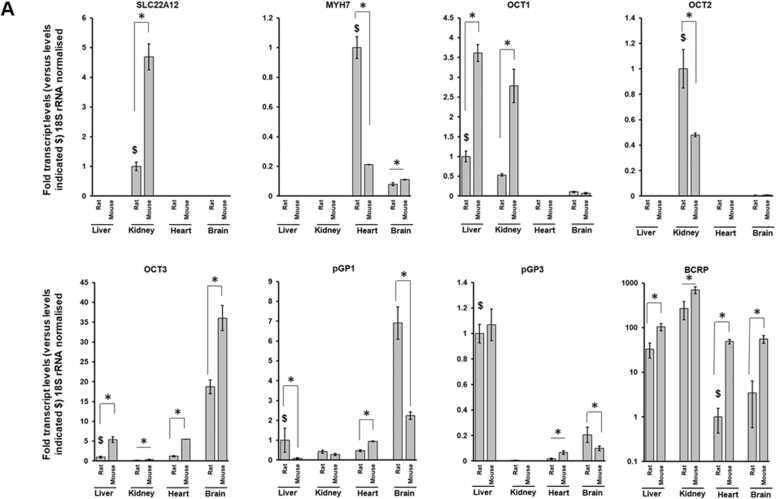

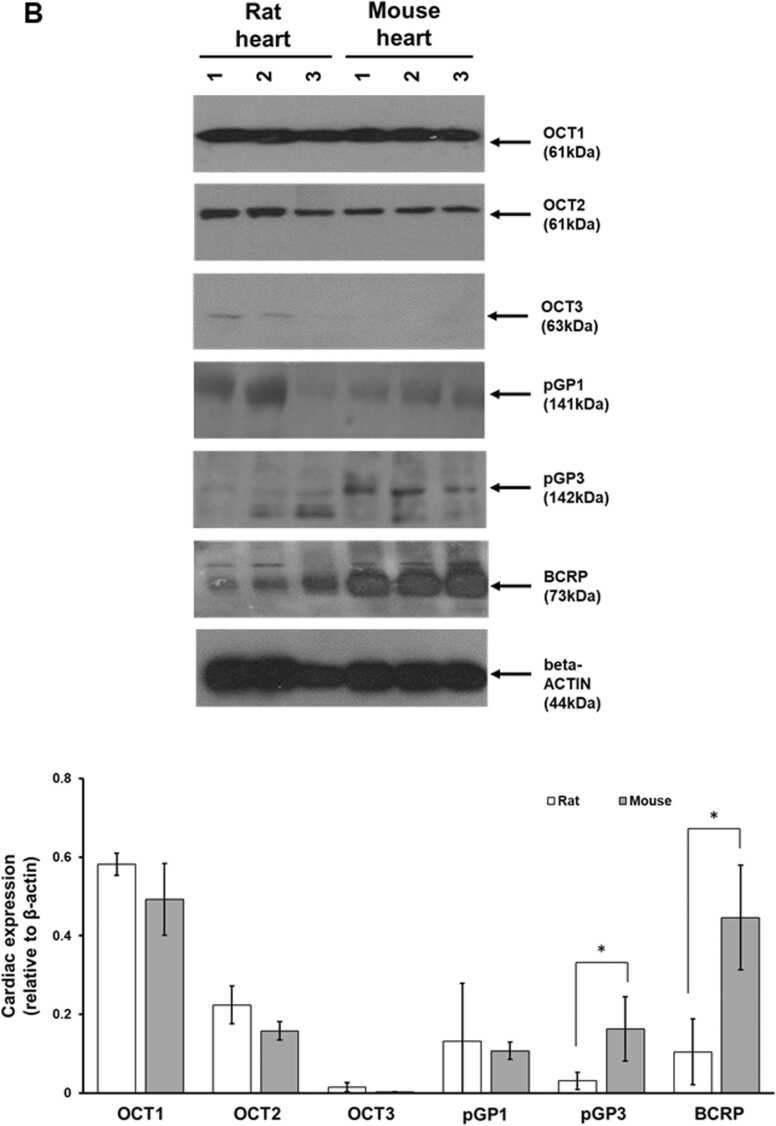
Mouse expresses higher levels of cardiac p-glycoprotein 3 and BCRP compared to rats. A, qRT-PCR quantification for the indicated transcript in rat and mouse tissues, expressed relative to the indicated normalised transcript indicated with $. Data are the mean and standard deviation tissue expression levels determined from 3 separate animals. *Statistically-significantly different fold (*18S* rRNA normalised) transcript level between rat and mouse using the Students T test (two tailed, p < 0.05). B, upper panel, Western blot for the indicated protein from 3 separate rat and mouse hearts; lower panel, relative expression levels of the indicated protein normalised to beta-actin. Full blot views are available in Figure S2. *Statistically-significantly different protein concentration (beta-actin normalised) between rat and mouse using the Students T test (two tailed, p < 0.05).

An examination of selected transporter mRNA transcript levels in heart and other tissues in rat and mouse by qRT-PCR indicates that Oct transcripts are broadly in the same range relative to *18 S* rRNA between rat and mouse (i.e. < 5 fold difference) (Fig. 6a). With regard to transporters facilitating excretion, the difference in relative expression of p-glycoprotein-1/*Abcb1b* (and the related p-glycoprotein 3/*Abcb4* transcript) between rat and mouse is even less marked (i.e. <3 fold) (Fig. 6a). In contrast, the relative expression of the *Bcrp* transcripts between rat and mouse was found to be 50-fold lower in rat. These differences in relative transcript levels between rat and mouse were broadly reflected in the levels of protein observed in heart tissues. Of note, the levels of BCRP protein in heart tissue was seen to be significantly higher when normalised to beta-actin levels (Fig. 6b).

**Fig. 7:**
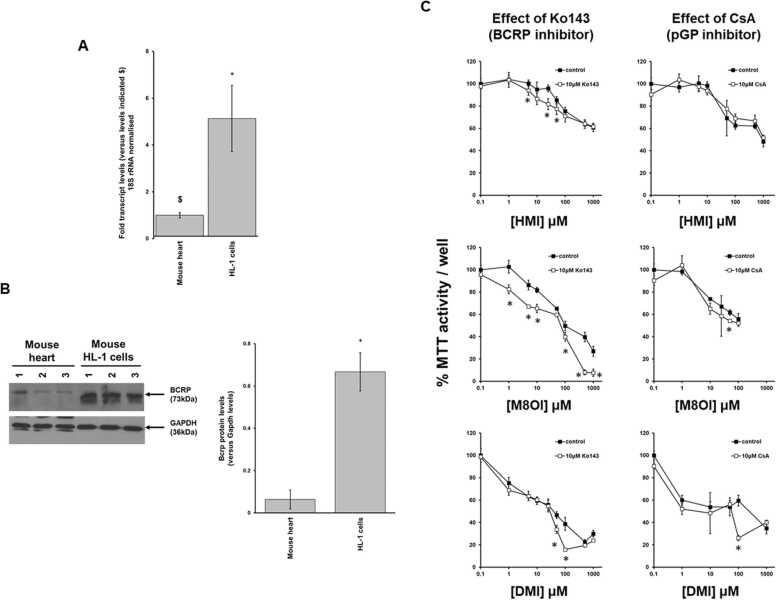
Mouse HL-1 cardiomyocytes express high levels of *Bcrp*/BCRP, are resistant to longer chain MIL toxicity and show increased sensitivity to longer chain MILs in the presence of the BCRP inhibitor Ko143. A, qRT-PCR quantification of *Bcrp* mRNA transcript in mouse heart and HL-1 cells. Data are the mean and standard deviation from 3 separate mouse hearts and 3 cultures of HL-1 cells. B, left panel, Western blot for the indicated protein from 3 separate mouse hearts and 3 cultures of HL-1 cells; right panel, expression levels of BCRP normalised to glyceraldehyde 3 phosphate dehydrogenase (GAPDH) levels. Note that the levels of immunoreactive beta-actin in HL-1 cells was very low and therefore could not be used to in quantification. The faster migrating protein in HL-1 cells is noted although the cause is not known but likely is dependent on altered glycosylation given that it is a membrane-associated protein. Full blot views are available in Figure S3. C, HL-1 cells were treated with increasing concentrations of the indicated MIL in the absence or presence of the indicated transporter inhibitor. MTT was determined 24 h later. Data are the mean and SD of 5individual determinations from the same experiment, typical of 3 separate experiments. *Significantly different (two tailed, p < 0.05) from control using the one-way analysis of variance (ANOVA) test followed by Bonferroni multiple comparison’s tests.

It was therefore hypothesised that BCRP may function to excrete MILs from the heart in mice (in contrast to rats, where p-glycoprotein-1 appears to play the most prominent role). To test this hypothesis, the toxicity of the longer chain MILs was examined in the mouse cardiomyocyte HL-1 cell line in the absence and presence of the BCRP inhibitor Ko143 or p-glycoproteins inhibitor cyclosporin A. The HL-1 cell line is a mouse proliferative atrial cardiomyocyte that originated from the SV40-transformed tumour cell line AT-1. HL-1 cells retain the ability to maintain a cardiac contractile characteristic upon serial passaging (Claycomb et al., 1998). Note that cyclosporin A inhibits both p-glycoprotein-1 and 3 (Kadmon et al., 1993, Kino et al., 1996, He and Liu, 2002, Elamiri et al., 2003). The HL-1 cell line expressed higher levels of *Bcrp* mRNA transcript (Fig. 7a) and BCRP protein (Fig. 7b) compared to mouse heart tissue, supporting its suitability as a model to test for the effects of BCRP function on an endpoint such as toxicity. Fig. 7c examines the toxicity of HMI, M8OI and DMI respectively in the absence and presence of the transporter inhibitors. It can be seen that HL-1 cells were relatively resistant to MILs. For example, the EC_50_ (effective concentration leading to a 50% reduction in MTT activity over 24 h) for DMI is 58 ± 4.67 μM (Fig. 7c) whereas over the same time period, the EC_50_ for DMI in B-13 cells was determined to be 2.2 ± 0.66 μM (Abdelghany et al., 2020). Identical experiments performed with HL-1 cells in the medium used for B-13 cells did not appreciably change the HL-1 cells resistance (data not included), indicating that a medium constituent(s) in the Claycomb medium used to culture HL-1 cells is not responsible for the resistance. The high levels of expression of BCRP in HL-1 cells may therefore at least in part be responsible for their resistance to longer chain MILs. In support of this, Fig. 7c in all cases indicate evidence that Ko143 exacerbated the toxicity of the longer chain MILs. However, the effects are relatively weak, suggesting that other unidentified factors likely also play a role in this in vitro model.

## 4 Discussion

To date, only limited data are publicly available regarding the adverse effects of MILs in mammalian systems which identify the kidney as a target organ. (Leitch et al., 2020b, Young et al., 2020; National Toxicology Program (NTP), 2022). The study herein identifies for the first time, a cardiac hazard associated with MILs, particularly with longer chain MILs. We demonstrate that MILs have the capacity to cause cardiomyocyte beat dysfunction and at higher concentrations cardiomyocyte death in rats.

Based on a comparative study in mammalian cells in vitro (Abdelghany et al., 2020), there is a trend for increased toxicity with increasing length of alkyl chain and this is also observed with neonatal cardiomyocytes in terms of both beat dysfunction and cell death. Of note, even with the least toxic MIL – EMI – a no adverse effect concentration (NOAC) for beat amplitude was 10 μM. For all other MILs, NOACs for both beat rate and beat amplitude were not identified, as they all produced effects at the lowest concentration examined of 10 μM. Future studies should examine the effects of lower concentrations of MILs in order to identify their NOACs. Based on in vitro studies with a variety of cell types, in terms of mechanism(s), cell death in response to MILs appears broadly to be associated with mitochondrial dysfunction, oxidative stress and apoptosis (Li et al., 2012, Jing et al., 2013; Li et al., 2015; Li et al., 2015; Probert et al., 2018; Ma et al., 2018; Li et al., 2020). Preliminary studies in HL-1 cells suggest a similar mechanism (unpublished observations).

Studies in rats also demonstrated that both sub-acute and sub-chronic exposure to longer chain MILs (HMI, M8OI and DMI) resulted in evidence of cardiac injury based on clinical chemistry endpoints and histopathological changes. Accordingly, the effects of MILs on rat cardiomyocytes in vitro may be relevant to the cardiac injury observed in rats in vivo. These studies represent the only examination of cardiac effects in mammals to date. Limited acute toxicity studies by others with M8OI using an intraperitoneal route of exposure did not examine heart tissue (Yu et al., 2008). The recently published NTP oral drinking water study on EMI and BMI in mouse and rat (up to 3 months exposure) performed a full histopathology on all control animals and all animals in the highest exposed groups, which included the heart (National Toxicology Program (NTP), 2022). In agreement with these studies, no exposure-related gross or histopathological changes were observed in male or female rats exposed to EMI and BMI.

As stated previously, cardiac affects were not observed in studies in which M8OI was administered to mice in vivo. From the extensive studies into drug efficacy and toxicity, it is widely acknowledged that the proteins transporting drugs into and out of cells are important determinants. Accordingly, altered drug transporter function, whether due to genetic polymorphisms, drug-drug interactions, or environmental factors such as dietary constituents, can result in unexpected toxicity (DeGorter et al., 2012). Data in this paper give some insight into this potential species difference. In mice, the longer chain MILs appear to be excreted from cells via the BCRP transporter whereas in rats, p-glycoprotein is the primary transporter of MILs. Given the high expression of BCRP in mouse compared to rat heart, there may be a more efficient removal of longer chain MILs from mouse cardiac tissue and hence reduced susceptibility.

OCT1 has been shown to mediate the uptake of M8OI in rat B-13 cells in vitro. OCT1 expression was found to be similar in rat and mouse heart (Figure 6b) and although this transporter may facilitate MIL uptake in the heart, it does not explain the apparent difference between rat and mouse and MIL cardiotoxicity. Expression of OCT3 was detectable, albeit low, in rat heart but was not detectable in mouse heart (Fig. 6b). OCT3 may therefore also be a determinant in MIL cardiotoxicity. However, this could not be tested because OCT3 is not expressed in rat B-13 cells (Hedya et al., 2023).

Of note, in all four of the tissues examined, mice express significantly higher levels of *Bcrp* mRNA transcripts compared to rats (with the greatest difference seen in heart), which might be reflected in more rapid clearance of the MILs in mice. To date, only limited toxicokinetics have been performed with long chain MILs. However, a detailed study with BMI indicates that the rates and routes of elimination were similar in male rats and female mice (86–95% of dose eliminated in 24 h). It is also shown that the systemic bioavailability of BMI is high, that tissue disposition and metabolism are negligible, and absorbed compound is extensively extracted by the kidney and eliminated in the urine as the parent compound (Sipes et al., 2008). These toxicokinetic features may explain the limited adverse effects of small chain MILs in vivo.

At the present time, it is uncertain which experimental species would be most reflective of the situation in man. In terms of actual risk to man, only limited conclusions can be drawn since there is limited information available on exposure (Leitch et al., 2020a). Smaller chain MILs are shown in this study to be relatively non-toxic. Smaller chain MILs are also more widely used although actual manufacturing/import levels in the EU are in some cases confidential (Leitch et al., 2020a). The MILs examined in this study are not declared to be used, to our knowledge, in food, medicines or cosmetics (Leitch et al., 2020a) and therefore if used in some way in processing, should only be present as a residual. It is likely that they are only used in industrial processes. Therefore, exposure to these MILs should be negligible and any health concern should be minimal.

However, there are indicators that some concern at the present time is warranted, even for the smaller, less toxic MILs. This is because the smaller chain MILs appear to be stable in the environment. For example, less than 10% of the dissolved organic carbon is lost after 28 days with EMI when incubated with activated sewage sludge (ECHA, 2022). Although larger chain MILs are subject to metabolism on the alkyl chain, there is no evidence, at the current time, to demonstrate full mineralisation. For example, 100% of DMI is metabolised from the parent after 21 days in aerobic sewage incubations but all products are limited to a variety of alkyl chain oxidations and modifications (Liwarska-Bizukojc et al., 2015). Of note, the methylimidazolium moiety appears to be environmentally stable. Accordingly, there may be a risk that methylimidazolium chemicals – either intact or as metabolites – accumulate over time. As water-soluble chemicals, they are also problematic to identify and quantify through traditional approaches. Finally, it has recently be determined that a low use MIL – M8OI – is detectable, albeit at low levels, in serum from humans (Leitch et al., 2021). No examination of the small chain/high use MILs has been undertaken.

MILs alone, therefore may represent a hazard in man in the future, particularly if use levels increase. The impact that MILs exposure has on sensitivity to cardiotoxic drugs, heart disease and other chronic diseases is unknown.

## CRediT authorship contribution statement

**Tarek Abdelghany**: Formal analysis, Investigation, Data curation. **Shireen Hedya**: Formal analysis, Investigation, Data curation. **Carol De Santis**: Formal analysis, Investigation, Data curation. **Sahar El-Rahman**: Formal analysis, Investigation, Data curation. **Jason Gill**: Funding acquisition, Writing − review & editing. **Noha Abdelkader**: Resources, Investigation, Writing − review & editing. **Matthew Wright**: Visualization, Supervision, Writing – original draft, Conceptualization, Methodology, Project administration, Funding acquisition.

## Declaration of Competing Interest

The authors declare that they have no known competing financial interests or personal relationships that could have appeared to influence the work reported in this paper.

## Data Availability

Data will be made available on request.
