# Supplementary material for: Potential for cardiac toxicity with methylimidazolium ionic liquids
Source: Ecotoxicol Environ Saf. 2023 Jan 1;249:114439. doi: 10.1016/j.ecoenv.2022.114439 (PMC10262066; doi:10.1016/j.ecoenv.2022.114439)
Supplement: Supplementary file 1 — Supplementary material. [file mmc1.docx]

**Supplementary Data: Potential for cardiac toxicity with methylimidazolium ionic liquids**

Tarek M. Abdelghany^2,1^ ▪ Shireen A. Hedya^2,1^ ▪ Carol De Santis^3^ ▪ Sahar S. El-Rahman^4^ ▪ Jason H. Gill^3^ ▪ Noha F. Abdelkader^2^ ▪ Matthew C. Wright^1,2^

^1^Institute Translational and Clinical Research, Level 4 Leech, Newcastle University, Newcastle Upon Tyne, United Kingdom NE2 4HH.

^2^Department of Pharmacology and Toxicology, Faculty of Pharmacy, Cairo University, Kasr El-Aini St., Cairo 11562, Egypt.

^3^School of Pharmacy, King George VI Building, Newcastle University, Newcastle Upon Tyne, United Kingdom NE2 4HH.

^4^Faculty of Veterinary Medicine, Cairo University, Giza, 12211, Egypt.

Corresponding author: Prof Matthew Wright, Translational and Clinical Research Institute, Level 4 Leech, Newcastle University, Newcastle Upon Tyne, United Kingdom NE24HH, tel (+44) 208 7094; fax (+44) 208 7745; email [m.c.wright@ncl.ac.uk](mailto:m.c.wright@ncl.ac.uk)

**Materials**

Anti-OCT1/SLC22A1 antibody (#ab181022), anti-OCT2/SLC22A2 (#ab170871), anti-OCT3/SLC22A3 (#ab183071), anti-p-glycoprotein-1/MDR1/ABCB1B (#ab235954), anti-p-glycoprotein-3/MDR2/ABCB4 and anti-BCRP/ ABCG2 (#ab207732) were purchased from Abcam (Cambridge, UK). The anti-beta-actin (# 60008-1-Ig) and anti-GAPDH (2118S) antibodies were purchased from Proteintech (Manchester, UK) and Cell Signaling Technology (Leiden, the Netherlands) respectively.

**Cell line culture and viability**

Thiazolyl blue tetrazolium bromide (MTT) reduction (as a proxy for cell viability) was determined following experimental treatments through addition of 350μM MTT to culture medium and incubation for 1 hour at 37^o^C in an humidified incubator. The medium was then discarded and cells treated with a fixed volume of isopropanol per well. After 10 minutes, absorbance was determined at 570 nm (with background absorbance at 690 nm also determined and reading subtracted from reading at 570 nm). Results are expressed as percentage absorbance relative to vehicle treated cells.

The effect of a transporter inhibitor on M8OI-dependent MTT reduction was used to screen for potential transporters of M8OI. Inhibitors of M8OI uptake would be expected to lead to relative increases in MTT reduction in the presence of toxic concentrations of M8OI (relative to M8OI-only treated cells). In contrast, inhibitors of M8OI excretion would be expected to lead to a decrease in M8OI-dependent MTT reduction in the presence of toxic concentrations of M8OI (relative to M8OI-only treated cells). The effect of any transporter inhibitor on MTT cell reduction alone was also examined.

Cells were therefore seeded into 24 well plates and cultured overnight. The medium was then renewed with 0.5mLs medium/well containing additionally a non-toxic concentration of a transporter inhibitor (or 0.1% (v/v) DMSO vehicle control). After 1 hour, the medium was replaced with inhibitor or DMSO vehicle, with or without a fixed concentration of M8OI estimated to cause between 40-60% loss of MTT reduction activity over the period of the experiment. MTT reduction was determined as outlined above after 24 hours and the percentage activity observed at any inhibitor concentration compared to their respective control (i.e. DMSO control for inhibitor-only or M8OI control for those cultures challenged with M8OI (and causing 40-60% reduction in MTT activity after 24 hours). Thus, for cells treated with this fixed concentration of M8OI, any dose-dependent change in MTT activity relative to M8OI only cells is taken as evidence that M8OI is a substrate of the targeted transporter. An increase in MTT activity with increasing transporter inhibitor is evidence that the transporter mediates M8OI uptake, whereas a decrease is evidence that the transporter mediates M8OI excretion.

**Supplementary Table 1. Primer sequences used to compare the relative transcript levels in rat and mouse tissues.**

| **Primer name** | **Primer sequence** | **Comments** |
| --- | --- | --- |
| rmOct1US | ACAGAGTTTGTCGGCTCTGG | 75bp fragment.  Rat and mouse have a single *Oct1* transcript. The mouse sequence was used to identify potential hybridisation sites. |
| rmOct1DS | CACTAGCCCCACTGTGAAGG |  |
|  |  |  |
| rmOct2US | GCTGAGCTGTACCCCACATA | 55bp fragment.  Rat has a single *Oct2* transcript, Mouse has 2 variants. The rat sequence was used to identify potential hybridisation sites. |
| rmOct2DS | TGGAGGAGCAGACAAGGACA |  |
|  |  |  |
| rmOct3US | GGGGCTTTTACCTTGGGCTA | 78bp fragment.  Rat and mouse have a single *Oct3* transcript. The mouse sequence was used to identify potential hybridisation sites. |
| rmOct3DS | GCCAACGCCGAAACAGGATA |  |
|  |  |  |
| rmAbcb1bUS | AAGATGGGCAAAAAGAGTAAAAAGGAG | 82bp fragment in rat; 85bp fragment in mouse. Rat and mouse have a single *Abcb1b* transcript (also called p-glycoprotein 1; *Mdr*1). The rat sequence was used to identify potential hybridisation sites. |
| rmAbcb1bDS | GCCAATCTGCATAGCGAAACATC |  |
|  |  |  |
| rmAbcb4US | TTGAACTAGGCAGCATCAGCA | 87bp fragment.  Rat and mouse have a single *Abcb4* transcript (also called p-glycoprotein 3; *Mdr2*). The rat sequence was used to identify potential hybridisation sites. |
| rmAbcb4DS | CAGAGTATCGGAACAGTGTCAAC |  |
|  |  |  |
| rmBcrpUS | CTTAATGCTATTCTGGGACCCACAG | 173bp fragment.  Rat and mouse have 1 reference (also called *Abcg2*) sequence and an X1 isoform sequence. The rat sequence was used to identify potential hybridisation sites with the shortest mouse variant 5 sequence. |
| rmBcrpDS | CCCATCACAACGTCATCTTGA |  |
|  |  |  |
| rmSlc22a12US | TGGCTGGGTTTACGACCACAG | 111bp fragment.  Rat and mouse have a single *Slc22a12* transcript. The mouse sequence was used to identify potential hybridisation sites. These transcripts are enriched in kidney tissue (Yu et al., 2014; Yue et al., 2014). |
| rmSlc22a12DS | CCAGCCAGGAAGATGGACTG |  |
|  |  |  |
| rmMyh7US | GTGAAGGGCATGAGGAAGAG | 80bp fragment.  Rat has a single *Myh7* transcript, mouse has 2 variants. The rat sequence was used to identify potential hybridisation sites. These transcripts are enriched in heart and muscle tissues (Yu et al., 2014; Yue et al., 2014). |
| rmMyh7DS | CGCAGTAGGTTCTTCCTGTCT |  |
|  |  |  |
| rm18SrRNAUS | CCCGAAGCGTTTACTTTGAA | 136bp fragment.  These transcripts are present in all tissues and are used in the normalisation of transcripts between tissues. |
| rm18SrRNADS | CCCTCTTAATCATGGCCTCA |  |

**Supplementary Table 2. Summary of effects of MILs on rat neonatal cardiomyocytes.**

|  | **No Effect level** | | | | | | |
| --- | --- | --- | --- | --- | --- | --- | --- |
|  | **Beat rate** | **Beat rate reversibility** |  | **Beat amplitude** | **Beat amplitude reversibility** |  | **Cell Index** |
| **EMI** | >1mM | >1mM |  | 10µM | >1mM |  | >1mM |
| **BMI** | <10µM | 100µM |  | <10µM | <10µM |  | 100µM |
| **HMI** | 10µM | >1mM |  | <10µM | >1mM |  | >10µM |
| **M8OI** | <10µM | <10µM |  | <10µM | <10µM |  | <10µM |
| **DMI** | <10µM | <10µM |  | <10µM | <10µM |  | <10µM |

**Supplementary Figure 1.**


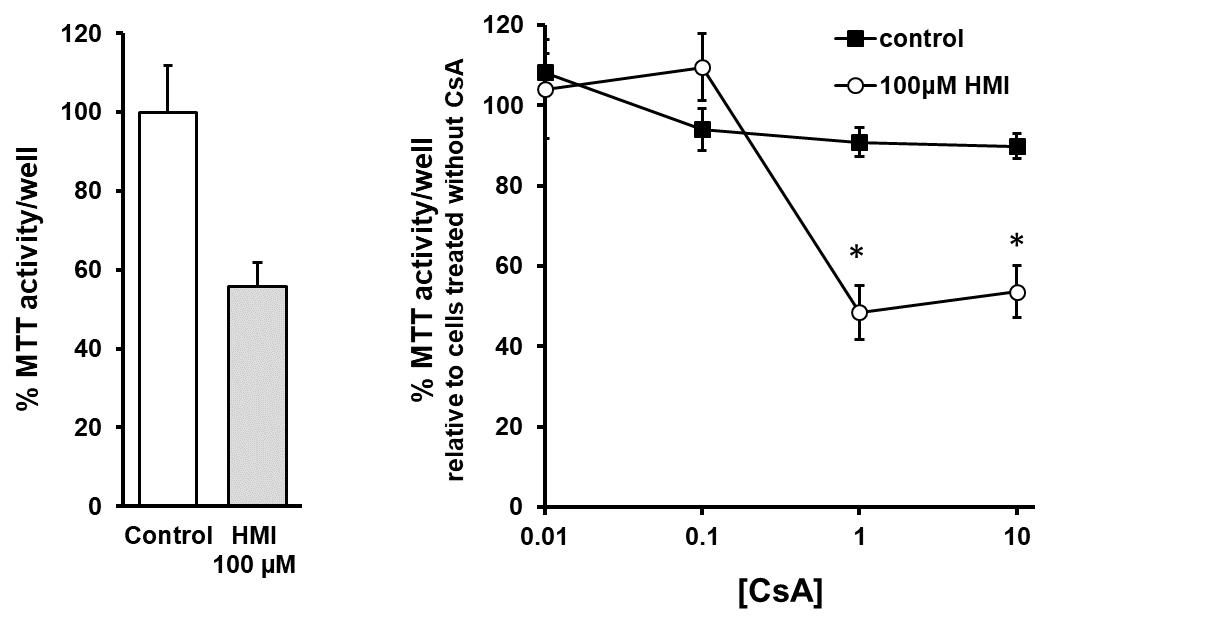


Supplementary Figure 1 demonstrates (with HMI) the typical effect seen when the p-glycoproteins inhibitor cyclosporin A is co-incubated with longer chain MILs in that the toxicity of the MIL is increased in rat B-13 cells

**Supplementary Table 3. Long chain MILs are excreted via p-glycoprotein in B-13 cells**

| **MIL** | **Transport substrate/inhibitor (target)** | **Effect on MIL toxicity** | **Comments** |
| --- | --- | --- | --- |
| **EMI** | amiodarone (pGp) | No effect | 1mM EMI alone resulted in 93 ± 5.9 % MTT reduction versus vehicle control treated cells. |
|  | CsA (pGp) | No effect |  |
|  | erythromycin (pGp) | No effect |  |
|  | imipramine (pGp) | No effect |  |
|  | quinidine (pGp) | No effect |  |
|  | Ko143 (Bcrp) | ↑toxicity |  |
|  |  |  |  |
| **BMI** | amiodarone (pGp) | No effect | 1mM BMI alone resulted in 59 ± 6.2 % MTT reduction versus vehicle control treated cells. |
|  | CsA (pGp) | No clear effect |  |
|  | erythromycin (pGp) | No effect |  |
|  | imipramine (pGp) | No effect |  |
|  | quinidine (pGp) | No effect |  |
|  | Ko143 (Bcrp) | No effect |  |
|  |  |  |  |
| **HMI** | amiodarone (pGp) | ↑toxicity | 100μM HMI alone resulted in 51 ± 5.8 % MTT reduction versus vehicle control treated cells. |
|  | CsA (pGp) | ↑toxicity |  |
|  | erythromycin (pGp) | ↑toxicity |  |
|  | imipramine (pGp) | ↑toxicity |  |
|  | quinidine (pGp) | ↑toxicity |  |
|  | Ko143 (Bcrp) | No effect |  |
|  |  |  |  |
| **M8OI** | amiodarone (pGp) | ↑toxicity | 8μM M8OI alone resulted in 67 ± 3.6 % MTT reduction versus vehicle control treated cells. Data taken from Hedya et al (2023). |
|  | CsA (pGp) | ↑toxicity |  |
|  | erythromycin (pGp) | ↑toxicity |  |
|  | imipramine (pGp) | ↑toxicity |  |
|  | quinidine (pGp) | ↑toxicity |  |
|  | Ko143 (Bcrp) | No effect |  |
|  |  |  |  |
| **DMI** | amiodarone (pGp) | No effect | 1μM DMI alone resulted in 59 ± 3.9 % MTT reduction versus vehicle control treated cells. |
|  | CsA (pGp) | ↑toxicity |  |
|  | erythromycin (pGp) | ↑toxicity |  |
|  | imipramine (pGp) | No effect |  |
|  | quinidine (pGp) | No effect |  |
|  | Ko143 (Bcrp) | No effect |  |

To determine whether p-glycoprotein and/or BCRP are involved to a significant degree in MIL excretion, B-13 cells were treated with a moderately toxic concentration of each MIL and the effect of co-treatment with substrates and inhibitors of p-glycoprotein and BCRP examined. MTT reduction was employed as a proxy for adverse effects/toxic effects.

**Supplementary Figure 2.** Full blot views for Figure 6b.


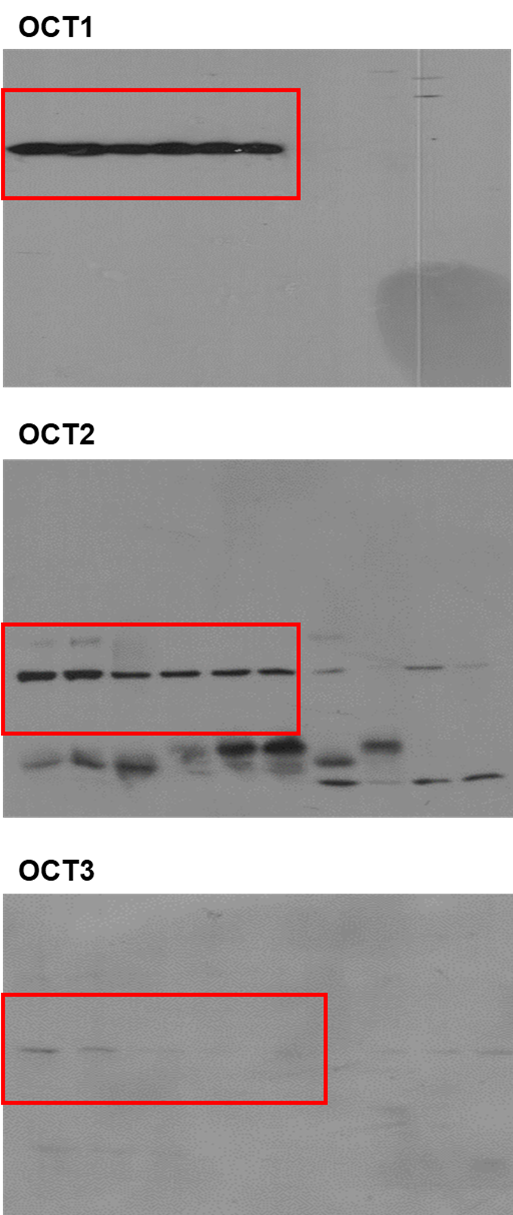


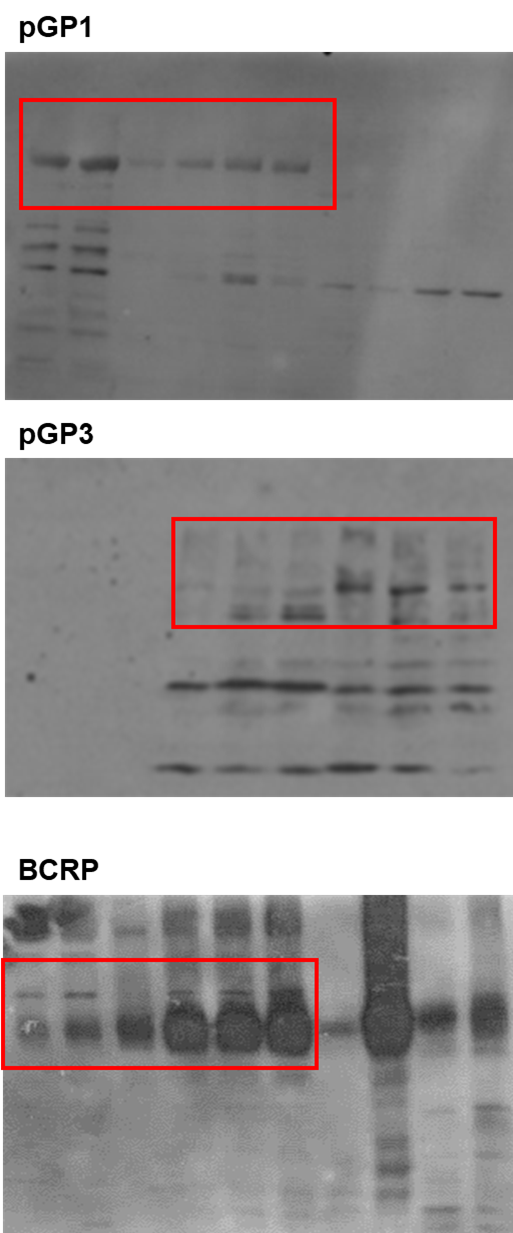


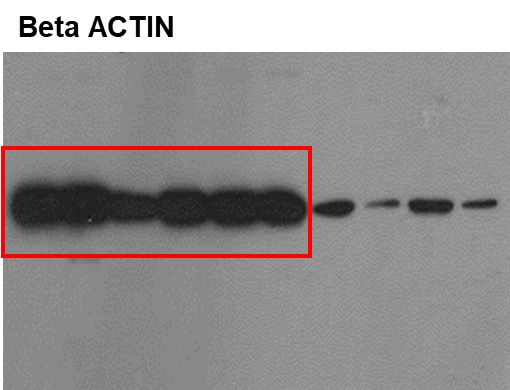


For each blot, the red square indicates the approximate region selected for presentation in the paper.

**Supplementary Figure 3.** Full blot views for Figure 7b.


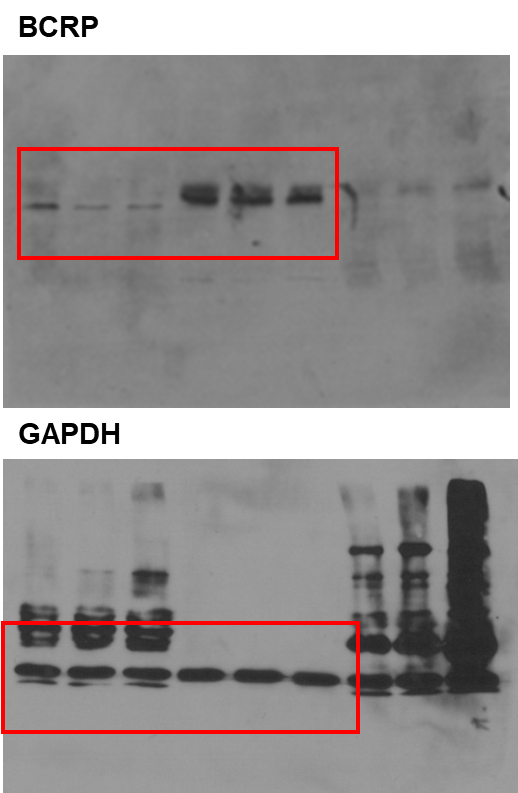


For each blot, the red square indicates the approximate region selected for presentation in the paper.

**References**

Yu Y, Fuscoe JC, Zhao C, et al (2014) A rat RNA-Seq transcriptomic BodyMap across 11 organs and 4 developmental stages. Nat Commun. 5: 3230. <https://doi.org/10.1038/ncomms4230>

Yue F, Cheng Y, Breschi A et al (2014) A comparative encyclopedia of DNA elements in the mouse genome. Nature 515: 355-64. <https://doi.org/10.1038/nature13992>
